# Clinical Network for Big Data and Personalized Health: Study Protocol and Preliminary Results

**DOI:** 10.3390/ijerph19116365

**Published:** 2022-05-24

**Authors:** Simona Esposito, Sabatino Orlandi, Sara Magnacca, Amalia De Curtis, Alessandro Gialluisi, Licia Iacoviello, on behalf of The Neuromed Clinical Network Big Data and Personalised Health Investigators

**Affiliations:** 1 Department of Epidemiology and Prevention, Istituto di Ricovero e Cura a Carattere Scientifico (IRCCS), Neuromed, 86077 Pozzilli, Italy; simona.esposito@moli-sani.org (S.E.); sabatino787@gmail.com (S.O.); amalia.decurtis@moli-sani.org (A.D.C.); alessandro.gialluisi@moli-sani.org (A.G.); 2 Mediterranea Cardiocentro, 80122 Napoli, Italy; sara.magnacca@moli-sani.org; 3 Department of Medicine and Surgery, Research Center in Epidemiology and Preventive Medicine (EPIMED), University of Insubria, 21100 Varese, Italy

**Keywords:** electronical health records, prevention, personalized medicine

## Abstract

The use of secondary hospital-based clinical data and electronical health records (EHR) represent a cost-efficient alternative to investigate chronic conditions. We present the Clinical Network Big Data and Personalised Health project, which collects EHRs for patients accessing hospitals in Central-Southern Italy, through an integrated digital platform to create a digital hub for the collection, management and analysis of personal, clinical and environmental information for patients, associated with a biobank to perform multi-omic analyses. A total of 12,864 participants (61.7% women, mean age 52.6 ± 17.6 years) signed a written informed consent to allow access to their EHRs. The majority of hospital access was in obstetrics and gynaecology (36.3%), while the main reason for hospitalization was represented by diseases of the circulatory system (21.2%). Participants had a secondary education (63.5%), were mostly retired (25.45%), reported low levels of physical activity (59.6%), had low adherence to the Mediterranean diet and were smokers (30.2%). A large percentage (35.8%) were overweight and the prevalence of hypertension, diabetes and hyperlipidemia was 36.4%, 11.1% and 19.6%, respectively. Blood samples were retrieved for 8686 patients (67.5%). This project is aimed at creating a digital hub for the collection, management and analysis of personal, clinical, diagnostic and environmental information for patients, and is associated with a biobank to perform multi-omic analyses.

## 1. Introduction

Many common diseases, such as cardiovascular diseases (CVD), asthma, arthritis, cancer, diabetes, obesity, and neurodegenerative and neuropsychiatric disorders, represent a notable societal burden in terms of health costs and productivity [1]. The Global Burden of Diseases collaboration revealed that a large number of deaths are known to be caused by high body-mass index, including cardiovascular diseases, neoplasms, dementia, asthma, hepatobiliary diseases, as well as diabetes and kidney diseases [2]. In particular, CVD and cancer are responsible for three out of four deaths in Western countries and for most of the morbidity and disabilities [3]. Collections of biological materials, or biobanks, together with the clinical information associated with the individual, represent an indispensable tool to identify risk factors-be they genetic or environmental-at the basis of the main health conditions [4]. Moreover, these modern tools allow for the translation of biomedical research into improved care efficacy, reduced healthcare costs and increased productivity thanks to a healthier population [5]. Also, the identification of molecular pathways involved in disease initiation or progression may lead to the discovery of new therapeutic targets, specific for each patient, hence to the development of personalized medicine approaches [6]. For these reasons, biobanks are of fundamental importance for epidemiological research, be it population- or disease-based [7]. Personalized medicine is based on the assumption that patients with the same disease are different from one another and thus respond differently to the same treatment [8]. Therefore, this approach is aimed at enabling clinicians to prescribe the right medicine to the right patient at the right time, with maximum efficacy and minimal toxicity [9]. Within this context, the real challenge is represented by the accessibility to volumes of large, complex, linkable information relevant to each patient [10], which often goes under the umbrella definition of Health Big Data [11,12]. This term covers a vast range of data sources, including medical, environmental, socioeconomic, geographic, and social media information, as well as genomic and other “omic” data [13]. Among these data, electronic health records (EHRs), defined as a systematized collection of patient and population electronically stored health information in a digital format [14], are of utter importance in the field of personalized medicine thanks to the large availability within modern clinical settings, and their use has increased in the last decade [15,16]. EHRs include a vast range of diverse patient-relevant data sources, including anamnestic information, diagnoses, anthropometric variables, laboratory tests and results, radiological images, multi-omic data and clinical notes of a different nature [17]. The use of EHRs substantially reduces medical error rates and health care costs and improves the quality of care [18], providing a rich source of data for research and favouring advance care planning (ACP), namely the process of planning for the future health care of subjects [19,20]. Also, EHRs represent useful resources in the development and application of medical Expert Systems (ERs), namely computer technologies developed to emulate human decision-making and to provide computerized clinical decision support to patients, clinicians and other expert domains, with the aim of improving healthcare delivery and organization. Indeed, ERs have been successfully applied in several instances related to clinical practice, including computer-assisted diagnosis (to suggest clinical diagnosis based on specific patient characteristics, signs and symptoms) and therapy (e.g., to suggest patient-tailored drug dosages), medication alert systems (e.g., to reduce adverse drug events) and patient-specific reminder systems (e.g., to improve compliance and efficacy of treatments) (see [21] for a comprehensive review). Overall, such ERs technologies have the potential to notably reduce medical error rates and to improve the efficacy of human and financial resources used in healthcare and prevention settings. This makes it very important to collect EHR data both in clinical and in epidemiological settings, warranting the need for the construction of appropriate data collection and elaboration infrastructures.

Here, we present the protocol and an ad interim descriptive analysis of data collected in the Clinical Network Big Data and Personalised Health Project, an initiative aimed at the creation of a digital platform for collection, management and analysis of personal, clinical, diagnostic and environmental data of patients admitted to twelve clinics and hospitals in Central-Southern Italy, relying on the IRCCS Neuromed hospital informatic and biobanking infrastructure. By implementing the storage and integration of biomedical data from very diverse sources into a unique platform, we aim to reach two complementary goals: (i) identify subtypes and risk factors of common health conditions, and (ii) predict morbidity and hospitalization risk, mortality and patient flow across clinical units in the future. This latter aspect may help improve the management of resources in clinical settings and the quality of the healthcare provided.

## 2. Materials and Methods

### 2.1. Study Population

**Figure 1:**
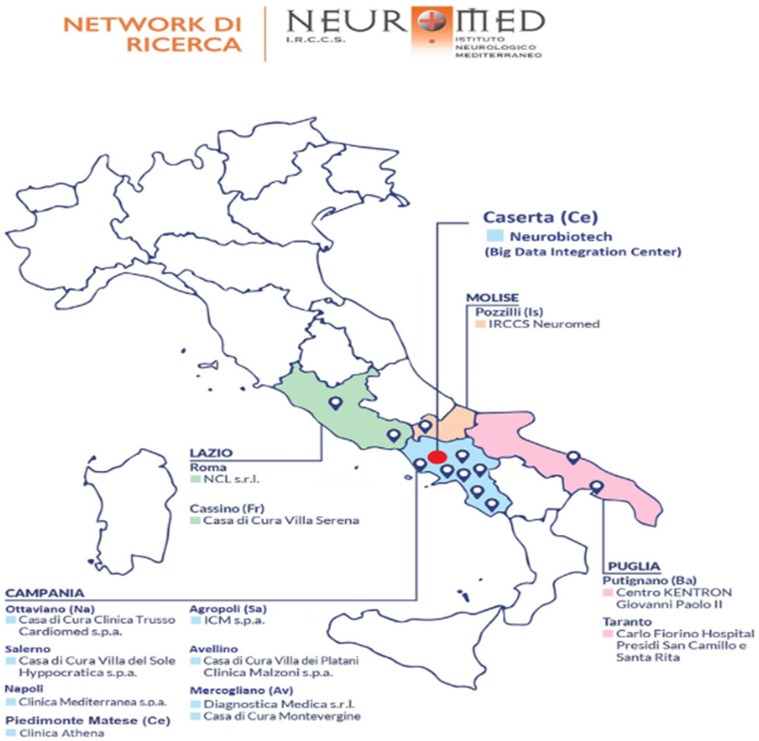
Geographical location of the hospitals/clinics involved in the project.

We performed a pilot analysis of 12,864 patients recruited from March 2019 to May 2021 from different clinics from Central and Southern Italy (see below). Inclusion criteria are:

- being hospitalized for at least 24 h in one of the involved clinics of the Neuromed group, which cover almost all regions of Central-Southern Italy: IRCCS Neuromed (Pozzilli, Isernia), Clinica Mediterranea (Napoli), Istituto Clinico Mediterraneo (Agropoli, Salerno), Villa del Sole (Salerno), Diagnostica Medica (Avellino), Clinica Malzoni (Avellino), Casa di cura Trusso (Ottaviano, Napoli), Neurological Centre of Latium (Roma), Villa Serena (Cassino, Frosinone), Carlo Fiorino Hospital (Taranto), Centro Giovanni Paolo II (Putignano, Bari), Clinica Athena (Piedimonte Matese, Caserta) (Figure 1).

Exclusion criteria are:

- being hospitalized for day surgery
- accessing an intensive care unit
- being under 18 years of age.

The study was authorized by the ethical committees of participating clinics, namely: Ethical Committee IRCCS NEUROMED, Ethical Committee CAMPANIA CENTRO ASL NA1, Ethical Committee CAMPANIA NORD, Ethical Committee CAMPANIA SUD ASL NA3, Ethical Committee CAMPANIA NORD AZIENDALE ASL AVELLINO, Ethical Committee LAZIO 2, Ethical Committee INDIPENDENTE, Ethical Committee AZIENDA SANITARIA LOCALE BR-COMITATO ETICO INDIPENDENTE DI ETICA MEDICA. The aims are to recruit a large hospitalized population that will allow us to evaluate which could be the risk factors common to the main diseases and to compare the lifestyles of patients with different pathologies. All the participants provided written informed consent, which allows us to obtain personal and clinical information (see below), to retrieve residual biological samples to be stored in a biobank and used for future omics analyses, and to perform a follow-up to monitor patient health status. To obtain personal information, cause/s of hospitalization and final diagnosis, every month we obtained patients records from an internal health data management and integration system (called Novamed^®^), a centralized platform including all biomedical and clinical data of interest for any patient accessing hospitals involved in the collaboration. Upon acceptance, questionnaires are provided to the patient to be filled in during the hospital stay and handled back to the local administrator (one or two for each recruiting centre). In the final phase of the project, all the data collected will be sent to a central data storage and elaboration informatic platform physically located in the Neurobiotech research centre (see Figure 1).

### 2.2. Questionnaires 

The adherence score to the Mediterranean diet is assessed through the MEDI-LITE score validated by Sofi and colleagues [22], testing the intake of nine food items and ranging between 0 and 18, with higher scores indicating higher adherence to the Mediterranean diet. To evaluate adherence to the Mediterranean diet, we categorized the population into tertiles of MEDI-LITE score (first tertile: 2–10 points; second tertile: 11 points; third tertile: 12–18 points). Patients were asked additional lifestyle information, e.g., about the type of water they drink (tap, in plastic or in glass bottle), smoking status (ever, past and current smokers, no of cigarettes per day and years of smoking), level of physical activity both in leisure time and during working hours, and habits of use with regard to cordless or mobile phones (no of hours per day).

Quality and duration of sleep was assessed through self-report, as well as whether this was continuous or interrupted, and if patients worked on night shifts (and, if so, how many times per week). Socioeconomic status (SES) was also assessed through investigating marital status (married/living in a couple or de facto relationship, separated/divorced, single, widowed), education level accomplished (lower, upper secondary and postsecondary education) and working class (student, manual, non-manual, professional and managerial worker, housewife, retired, unemployed). For women, information on menopausal status was also collected, as well as the number of pregnancies during the course of life.

Prevalent chronic health conditions like hypertension, diabetes and hypercholesterolemia are defined by reporting current pharmacological treatments for one or more of these diseases. Height and weight were self-reported, and body mass index (BMI) was calculated as kg/m² and then grouped into three categories as under/normal weight (<25), overweight (≥25 to <30), or obese (≥30). Biometric information like systolic/diastolic blood pressure and heart rate was also measured.

### 2.3. Biological Samples and Biobanking

For each participant, EDTA (Ethylenediaminetetraacetic acid) waste blood samples resulting from routine biochemical analyses were collected within each clinical centre involved in the project. Biological samples were transported through a weekly shuttle service from clinics to the Neuromed Biobanking Center in Pozzilli (IS). Here, the blood was centrifuged at 3000 rpm for 20 min at room temperature, and buffy coats were collected and stored in tanks with liquid nitrogen at −196 °C (within seven days from blood draw), as well as plasma aliquots (within two days from the blood draw). The Neuromed Biobanking Center is a research facility devoted to the collection, storage and redistribution of biological materials and a related database, managed according to standardized operating procedures, with ethical, legal and societal criteria established at regional, national and international levels. The Neuromed Biobanking Center operates according to the national and international guidelines for recognition and accreditation of biobanks (2006 report of the Comitato Nazionale per la Biosicurezza e le Biotecnologie of the Presidenza del Consiglio dei Ministri, modified in 2008). It is officially acknowledged by the European Infrastructure of Biobanks and Biomolecular Resources (BBMRI-ERIC) and by its Italian node BBMRI.it (Partner Charter Document, Milan, 25 May 2015). At the regional level, the Neuromed hospital has been recognized as an institution allowed to biobank “human cells, tissues and DNA” (Molise Regional Council resolution n.615, 24 November 2014). The biobanking infrastructure covers more than 500 square meters localized within the Neuromed Technology Park, and includes twelve freezers (T = −80 °C) and six liquid nitrogen tanks (T = −196 °C), which are connected to an external container of liquid nitrogen through appropriate distribution pipes, as well as to a nitrogen supply for back-up. Each sample stored in the biobank is identified by an alphanumeric code which allows for the exact localization in the containers and is connected, in an anonymous way, to each study database and related information. 

All donors of biological specimens stored in the biobank sign a study-specific informed consent, which was previously authorized by the institution’s ethical committee. Personal data of donors are stored according to the security rules for informatic archives (D.lgs 196/03) and of the privacy protection rules for data handling (GDPR, 95/46/CE).

### 2.4. Alternative Strategies during COVID-19 Pandemic

In February 2020, the spread of the COVID-19 epidemic in Italy began, and a few days later the World Health Organization (WHO) declared a pandemic [23]. On 9 March, Italian authorities declared a lockdown so as to hinder the spread of the disease by introducing such measures as social distancing. This notably affected the project, since all non-urgent hospitalizations and routine medical tests were suspended to help tackle the clinical emergency. Keeping in mind the potential psychological, health and lifestyle effects that social isolation may have on participants and the high likelihood that at least part of our participants may have or have been affected by SARS-CoV-2 infection during the first and following epidemic waves, we created an ad hoc questionnaire to assess these aspects. Patients accessing our clinics since October 2020 were then asked questions assessing how their lifestyle, psychological and socioeconomic status changed following the lockdown phase, whether they were diagnosed and or tested for SARS-CoV-2 infection, and if they presented potential (neurological) symptoms after COVID-19. Overall, 4280 participants had answered the COVID-19 section of the questionnaire as of 31 October 2021. Moreover, for a subset of 401 voluntary participants, we also have results of serum IgG and IgM antibody titres against SARS-CoV-2 antigens along with the residual serum samples for potential future analyses.

## 3. Results

12,864 participants (61.7% women) signed written informed consent to allow access to their EHRs, and were therefore analysed in the pilot study (mean (SD) age 52.6 ± 17.6 years). Of these, 739 patients accessed clinics of the network two or more times and 6036 unique patients (64.2% women) filled in questionnaires. Blood samples were retrieved for 8686 participants.

**Figure 2:**
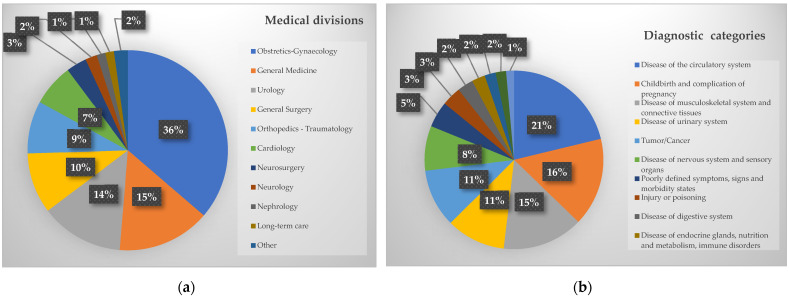
Distribution of participants to the project by (**a**) medical division and (**b**) diagnostic category.

Figure 2 reports the distribution of participants by hospital division (Figure 2a) and main diagnostic category (Figure 2b). The majority of hospital admissions were in obstetrics and gynaecology (36.3%), general medicine (15.0%) and urology (13.4%). The main reasons for hospitalization were diseases of the circulatory system (21.2%), childbirth, and complications of pregnancy (16.0%), and diseases of the musculoskeletal system and connective tissue (14.6%).

Participants were prevalently married or living in a couple or de facto relationship (74.8%), held an upper secondary education title (63.5%), were mostly retired (25.4%) or housewives (21.3%), and were mostly working in the agri-food (8.2%), textile (2.9%) and engineering (2.8%) sectors (Table 1).

The majority of participants (40.0%) showed a BMI lower than 25, while 35.8% were overweight (25 ≤ BMI < 30) and 22.0% were obese (BMI ≥ 30). Mostly, they managed to climb stairs without any difficulty (48.6%) and did the usual housework autonomously (54.9%). Most of them practiced low levels of physical activity, both in their spare time (59.6%) and during working hours (42.0%).

The majority of subjects drank water in plastic bottles (80.0%) and mainly reported a low adherence to the Mediterranean diet (35.6%). Just over 30% of participants were current smokers, while 22.5% were previous smokers. These subjects smoked an average of 15 cigarettes per day and had been smoking for 13.4 years on average.

Participants had a prevalently sedentary job (59.6%) with low levels of responsibility and mental stress (39.5%), and mainly reported their sleep to be quiet (76.1%) and lasting for six to seven h/day (40.6%), in line with the majority of the participants not working on night shifts (55.2%). Most used their mobile phone for less than two h/day (43.0%; see Table 2).

The majority of participants were free from hypertension (62.6%), diabetes (87.6%) and hyperlipidemia (79.1%). Most of the women were not in menopausal status (54.9%), and the average number of pregnancies per woman was 2.0 ± 1.6 (Table 3).

## 4. Discussion

In this manuscript, we provide an overview of a project designed for the collection, management, integration and analysis of health big data from a network of 12 clinics and hospitals in Central-Southern Italy, the Clinical Network Big Data and Personalised Health Project. To our knowledge, this represents one of the first large-scale initiatives of this kind in Italy, both in the private and in the public healthcare sector, and probably the first one in Southern Italy. Indeed, in spite of its renowned national healthcare system in terms of human resources and quality of care, Italy still suffers from a longstanding delay in collecting, organizing and exploiting health big data, in contrast with other large-scale and established initiatives in Western countries which are supported by national healthcare systems, e.g., in the UK [24].

In the Clinical Network Big Data and Personalised Health Project, we added, to the collection of EHRs from clinics/hospitals, further assessments on sociodemographic and lifestyle characteristics of the patients. Indeed, patient information which is not deemed as strictly relevant for the diagnosis is often overlooked, e.g., social history [25]. Moreover, we are working towards interpretation of hidden patterns underlying EHR data, both in terms of patients subtyping within specific diseases and in terms of prediction of the risk of re-hospitalization or patient flows across divisions, although the latter (supervised) analysis will be feasible only when a higher number of repeated events will be available in the cohort, it is worth to underline that both these complementary strategies will allow us to improve the quality and cost-efficacy of care [18], which represents the main goal of personalized medicine. For both of the above-mentioned ends of our initiative, the organization of EHRs into a data infrastructure is of utter importance. The first data collected allowed us to take a snapshot of the hospitalized population of Southern Italy, revealing interesting insights, like an average adherence to the Mediterranean diet. This evidence is in line with previous reports in the general Italian population [26], although these studies are not directly comparable since the Mediterranean diet was measured through different scales. Also, a preliminary analysis of the data revealed a notable prevalence of physiological deliveries within our cohort, which triggered a different project on the construction and follow-up of a longitudinal study of trios to investigate how the parental exposome may influence developmental outcomes in children. Other similar initiatives that focus on specific diseases (e.g., CVD) and conditions (e.g., obesity) are being planned and may stem from the current project.

### 4.1. Strengths of the Project

Beyond the obvious strengths of a large-scale multi-centre EHR-based study, our project is peculiar for a number of reasons. First, this is deployed within a Mediterranean population, with relatively homogenous cultural and lifestyle habits, but quite diverse nutritional habits across regions [27]. Second, a peculiar genetic background of the population from Southern Italy, where continuous gene flow along the centuries resulted in remarkable genetic variability [28], makes it even more interesting to investigate the susceptibility to complex disorders. Furthermore, the present study may serve as a substrate to investigate population genetic history of the country at a fine-grained resolution. Indeed, the genetic history of Southern Italy is characterized by a generally high degree of outbreeding, except for a few linguistic and genetic isolates [29,30,31], and notable contaminations from both Northern Africa and Eastern Europe over the centuries [32]. Moreover, to the best of our knowledge, other datasets are specific for one disease, e.g., heart failure [33] or viral infection [34], while our study involved a lot of different chronic disorders or physiological conditions (e.g., pregnancy) in a large population from Southern Italy. Additionally, our database will take into consideration the follow-up for each patient involved in the project and we foresee the ability to periodically update the dataset as the sample size increases. Finally, the diversity of biological samples collected may help analyse specific markers and metabolites retrospectively under hypothesis-driven approaches.

### 4.2. Potential Limitations

The potential hindrances that we may encounter during the course of the project and in the following data analysis are of both a logistic and statistical nature. First, the current lack of follow-up data on mortality outcomes represents a notable limitation at present, since it does not allow for the modelling of algorithms predicting incident death risks. However, we are planning to ask public authorities for access to the national mortality registry (Registro Nominativo Cause Morte, ReNCaM). The same applies for regional hospitalization registries of all the regions where our clinics/hospitals are located, although in this case the high fidelity of patients increases the likelihood that they get hospitalized in our centres, and this partly reduces this bias. Also, it is worth underlining that we are presently working with a large data goal of collecting massive volumes of data at a fast pace, without taking into account a specific study design. Second, the low recruitment and answer rate for some population strata, which is especially pronounced for older, less educated and low SES subjects, may introduce biases due to missing not at random (MNAR) data patterns. However, modern data imputation techniques like multiple imputation allow for the reduction of this bias, notably increasing the sample size and thereby its power [35]. Finally, although the current COVID-19 pandemic may further hamper recruitment, the project revealed a high resilience and adaptability to new clinical settings, even under stress conditions.

## 5. Conclusions

In conclusion, the Clinical Network Big Data and Personalised Health Project represents the first large hospitalized cohort in the South of Italy with the aims of implementing a platform that supports clinics, predicts the risk of morbidity, hospitalization and mortality, and improves the concept of personalized medicine.

In addition to the diversity of data collected within this framework, we are working towards integrating additional types of data, including both internal (neuroimaging, blood markers) and external exposomes (air pollution, drug prescriptions). Similar attempts were already carried out on a smaller scale within the institute, e.g., integrating L-Dopa dosages taken by Parkinson’s disease patients, anamnestic and clinical characteristics and genetic data to identify features predicting the risk of L-Dopa induced dyskinesia.

### Future Perspective

This represents a prominent example of how different types of EHRs may be used together in pharmacoepidemiology and translational medicine through a personalized approach. Also, to favour the exploitability of our data and in an open science perspective, we aim to make data available to the scientific community for potential collaborations (see Data Availability Statement) so as to speed up research in the field and improve the identification of novel risk factors for common diseases and data patterns predicting re-hospitalization risk.

## Figures and Tables

**Table 1:** Characteristics of the analysed cohort: sociodemographic characteristics.

Variables	*N* of Subjects (%)
Age groups (%)	
18–30	759 (12.6%)
31–50	2108 (34.9%)
51–70	2027 (33.6%)
71–90	1114 (18.5%)
90+	28 (0.5%)
Women (%)	3874 (64.2%)
Educational level (%)	
Up to lower school	929 (15.4%)
Upper secondary	3833 (63.5%)
Postsecondary education	1093 (18.1%)
Missing	181 (3.0%)
Occupation (%)	
Student	134 (2.2%)
Manual	1374 (22.8%)
Non-manual	779 (12.9%)
Specialized/management	398 (6.6%)
Housewife	1287 (21.3%)
Retired	1536 (25.4%)
Unemployed	391 (6.5%)
Do not wish to answer	66 (1.1%)
Missing	71 (1.2%)
Prevalent occupation (%)	
Agri-food	498 (8.2%)
Textile	175 (2.9%)
Engineering	167 (2.8%)
Chemical/pharmaceutical	122 (2.0%)
Extractive	6 (0.1%)
Electronics	56 (0.9%)
Construction	157 (2.6%)
Metallurgic	39 (0.6%)
Other	3405 (56.4%)
Missing	1411 (23.4%)
Marital status (%)	
Married/living in a couple or de facto relationship	4514 (74.8%)
Separated/divorced	300 (5.0%)
Single	756 (12.5%)
Widowed	422 (7.0%)
Missing	44 (0.7%)

**Table 2:** Characteristics of the analysed cohort: lifestyles and proxy measures.

Variables	*N* of Subjects (%)
Mediterranean diet (%)	
Low adherence (2 to 10)	2150 (35.6%)
Average adherence (11)	1389 (23.0%)
High adherence (12 to 18)	2105 (34.9%)
Missing	392 (6.5%)
Type of water (%)	
Plastic bottles	4828 (80.0%)
Glass bottles	280 (4.6%)
Tap water	698 (11.5%)
Missing	233 (3.9%)
Smoking status (%)	
Yes	1824 (30.2%)
No	2836 (47.0%)
Former	1356 (22.5%)
Missing	20 (0.3%)
Hours spent with mobile phone (%)	
<2 h	2596 (43.0%)
2–4 h	2284 (37.8%)
5–14 h	815 (13.5%)
>15 h	92 (1.5%)
Missing	249 (4.1%)
Hours spent with cordless phone (%)	
<2 h	2417 (40.0%)
2–4 h	149 (2.5%)
5–14 h	27 (0.4%)
>15 h	12 (0.2%)
Missing	3431 (56.8%)
Sleeping with phone nearby (%)	
Yes	2705 (44.8%)
No	3197 (53.0%)
Missing	134 (2.2%)
Physically active lifestyle (%)	
Yes	3451 (57.2%)
No	2461 (40.8%)
Missing	124 (2.0%)
Body mass index (%)	
Under/normal weight (<25 kg/m²)	2415 (40.0%)
Overweight (≥25, <30 kg/m²)	2164 (35.8%)
Obese (≥30 kg/m²)	1327 (22.0%)
Missing	132 (2.2%)
Quality of sleep (%)	
< 4 h	251 (4.1%)
5–6 h	1813 (30.0%)
6–7 h	2449 (40.6%)
7–8 h	1278 (21.2%)
> 8 h	188 (3.1%)
Missing	10 (0.2%)

**Table 3:** Characteristics of the analysed cohort: physiological and pathological conditions.

Variables	*N* of Subjects (%)
Number of pregnancies (median; SD)	(2; 1.6)
Menopausal status (%)	
Yes	1686 (43.5%)
No	2128 (54.9%)
Missing	60 (1.5%)
Hypertension (%)	
Yes	2195 (36.4%)
No	3779 (62.6%)
Do not wish to answer	11 (0.2%)
Missing	51 (0.8%)
Diabetes (%)	
Yes	671 (11.1%)
No	5287 (87.6%)
Do not wish to answer	19 (0.3%)
Missing	59 (1.0%)
Hyperlipidaemia (%)	
Yes	1183 (19.6%)
No	4773 (79.1%)
Do not wish to answer	19 (0.3%)
Missing	61 (1.0%)
Systolic blood pressure (mmHg) (median; SD)	(121.8; 13.1)
Min	60
Max	225
Diastolic blood pressure (mmHg) (median; SD)	(74.0; 9.0)
Min	20
Max	160
Heart rate (bpm) (median; SD)	(73.2; 7.8)
Min	34
Max	180

## Data Availability

Anonymized data underlying this manuscript will be made available upon reasonable request by interested collaborators and approval of a scientific proposal by the corresponding author, Licia Iacoviello.

## Appendix A

**Clinical Network Big Data and Personalised Health Project Study Investigators**
**Principal Investigators**: Licia Iacoviello, MD, PhD, (IRCCS Neuromed, Pozzilli and Università dell’Insubria, Varese, Italy)
**Steering Committee**: Giovanni de Gaetano, Maria Benedetta Donati, Chiara Cerletti, Alessandro Gialluisi, Amalia De Curtis, Simona Costanzo, Marialaura Bonaccio (Department of Epidemiology and Prevention, IRCCS Neuromed, Pozzilli), Augusto Di Castelnuovo (Mediterranea Cardiocentro, Napoli).
**Recruitment coordinator**: Simona Esposito (Department of Epidemiology and Prevention, IRCCS Neuromed).
**Neuromed Research Network**:
I.R.C.C.S. Neuromed, Pozzilli (Simona Esposito, Sabatino Orlandi)
  Clinica Malzoni, Avellino (Elena Bonanno, Maria Bianco, Annarita Vinciguerra)
  Diagnostica Medica, Avellino (Paola Bruni, Maria Bianco)
  N.C.L., Roma (Anna Campanella, Ida D’Anselmo, Edoardo Romoli, Pasquale Scognamiglio)
  Villa del Sole, Salerno (Maria Ceglia, Maria Grazia Caputo, Michelina Contangelo, Maria Rosaria Pandolfi, Giovanni Ricco)
  Clinica Athena, Piedimonte Matese (Maria Addolorata D’Abbraccio)
  Casa di Cura Trusso, Ottaviano (Alessandro Del Giudice, Camilla Esposito)
  Clinica Mediterranea, Napoli (Francesca De Micco)
  Carlo Fiorino Hospital, Taranto (Giovanni Pulito)
  I.C.M., Agropoli (Paola De Domenico, Aniello Formisano, Mariafiorella Tomasino)
  Centro Giovanni Paolo II, Putignano (Angela Vinci)
  Villa Serena, Cassino (Anna Izzo, Edoardo Romoli).
**Data analysis**: Simona Costanzo (Department of Epidemiology and Prevention, IRCCS Neuromed), Augusto Di Castelnuovo (Mediterranea Cardiocentro, Napoli), Alessandro Gialluisi (Department of Epidemiology and Prevention, IRCCS Neuromed), Sabatino Orlandi (Department of Epidemiology and Prevention, IRCCS Neuromed).
**Informatics**: Sabatino Orlandi (Department of Epidemiology and Prevention, IRCCS Neuromed)
**Biobanking**: Amalia De Curtis, Simona Esposito (Department of Epidemiology and Prevention, IRCCS Neuromed), Sara Magnacca (Mediterranea Cardiocentro, Napoli).
**Communication and Press Office**: Americo Bonanni (Department of Epidemiology and Prevention, IRCCS Neuromed).
